# Supplementary material for: Dynamic relocalization of replication origins by Fkh1 requires execution of DDK function and Cdc45 loading at origins
Source: eLife. 2019 May 14;8:e45512. doi: 10.7554/eLife.45512 (PMC6533057; doi:10.7554/eLife.45512)
Supplement: Transparent reporting form [file elife-45512-transrepform.pdf]

eLIFE
1st Floor
24 Hills Road
Cambridge CB2 1JP, UK
P 01223 855340
W elifesciences.org
T @elife

eLife's transparent reporting form

We encourage authors to provide detailed information *within their submission* to facilitate the interpretation and replication of experiments. Authors can upload supporting documentation to indicate the use of appropriate reporting guidelines for health-related research (see EQUATOR Network), life science research (see the BioSharing Information Resource), or the ARRIVE guidelines for reporting work involving animal research. Where applicable, authors should refer to any relevant reporting standards documents in this form.

If you have any questions, please consult our Journal Policies and/or contact us: editorial@elifesciences.org.

Sample-size estimation

- You should state whether an appropriate sample size was computed when the study was being designed
- You should state the statistical method of sample size computation and any required assumptions
- If no explicit power analysis was used, you should describe how you decided what sample (replicate) size (number) to use

Please outline where this information can be found within the submission (e.g., sections or figure legends), or explain why this information doesn't apply to your submission:

For each imaging quantification (distance measurements), similar or larger cell number were used for each assay as in previous published paper (Heun and Gasser, *Science* 2001 and Heun et al, J. Cell Biol. 2001). Sample sizes and P values are indicated in figures. The raw imaging quantification (.xlsx) and statistical analysis results (.csv) are provided in Dryad under Folder: Data->Imaging quantification. Files were saved in according folder with Figure name.

The Dryad temporary review link is
https://urldefense.proofpoint.com/v2/url?u=https-3A__datadryad.org_review-3Fdoi-3Ddoi-3A10.5061_dryad.7bm444s&d=DwIFaQ&c=clK7kQUTWtAVEOVIgvi0NU5BOUHhpN0H8p7CSfnc_gI&r=OlW6DpfbaDct0y5Y2PHXOQ&m=hB4yH6sMvL88OKlDKYiTtmEQuacla2KiCg5cEuVXFKU&s=X64jSw_XU-kP2kt-g67tN3iGQz0x60UkR8IOM2WyB9s&e=

Replicates

- You should report how often each experiment was performed
- You should include a definition of biological versus technical replication
- The data obtained should be provided and sufficient information should be provided to indicate the number of independent biological and/or technical replicates
- If you encountered any outliers, you should describe how these were handled
- Criteria for exclusion/inclusion of data should be clearly stated

eLife Sciences Publications, Ltd is a limited liability non-profit non-stock corporation incorporated in the State of Delaware, USA, with company number 5030732, and is registered in the UK with company number FC030576 and branch number BR015634 at the address 1st Floor, 24 Hills Road, Cambridge CB2 1JP | August 2014

- High-throughput sequence data should be uploaded before submission, with a private link for reviewers provided (these are available from both GEO and ArrayExpress)

Please outline where this information can be found within the submission (e.g., sections or figure legends), or explain why this information doesn't apply to your submission:

Each experiment was repeated at least twice. For each imaging quantification (distance measurements), each individual cell represents a biological replicate and cell number (n) is indicated in the figure. For BrdU-IP-seq data, experiments were conducted by 3 technical replicates. With ChIP-seq data, 2 technical replicates were conducted. Outliers are included and indicated in figures (boxplots). For Figure 6, tracking data for 3 individual cells was removed because of movement of the nucleus; these are highlighted in red in the quantification excel sheet (in Dryad under Folder: Data->Imaging quantification->quantification for Fig6).

Statistical reporting

- Statistical analysis methods should be described and justified
- Raw data should be presented in figures whenever informative to do so (typically when N per group is less than 10)
- For each experiment, you should identify the statistical tests used, exact values of N, definitions of center, methods of multiple test correction, and dispersion and precision measures (e.g., mean, median, SD, SEM, confidence intervals; and, for the major substantive results, a measure of effect size (e.g., Pearson's r, Cohen's d)
- Report exact p-values wherever possible alongside the summary statistics and 95% confidence intervals. These should be reported for all key questions and not only when the p-value is less than 0.05.

Please outline where this information can be found within the submission (e.g., sections or figure legends), or explain why this information doesn't apply to your submission:

All the statistical analysis was described in the figure legends and results provided in the figures. The detailed statistical analysis results (.csv) are provided in the folder in Dryad.

(For large datasets, or papers with a very large number of statistical tests, you may upload a single table file with tests, Ns, etc., with reference to sections in the manuscript.)

Group allocation

- Indicate how samples were allocated into experimental groups (in the case of clinical studies, please specify allocation to treatment method); if randomization was used, please also state if restricted randomization was applied
- Indicate if masking was used during group allocation, data collection and/or data analysis

Please outline where this information can be found within the submission (e.g., sections or figure legends), or explain why this information doesn't apply to your submission:

Not applicable in this study

Additional data files ("source data")

- We encourage you to upload relevant additional data files, such as numerical data that are represented as a graph in a figure, or as a summary table
- Where provided, these should be in the most useful format, and they can be uploaded as "Source data" files linked to a main figure or table
- Include model definition files including the full list of parameters used
- Include code used for data analysis (e.g., R, MatLab)
- Avoid stating that data files are "available upon request"

Please indicate the figures or tables for which source data files have been provided:

Movies for Fig6, Sequencing data and transcript used in figures are available in Dryad. Movies are under folder: Data->Imaging quantification->Movies for Fig6. Sequencing data and transcript data were saved in folders with the corresponding name in Dryad. Detailed description for Sequencing data is available in Dryad.

eLife Sciences Publications, Ltd is a limited liability non-profit non-stock corporation incorporated in the State of Delaware, USA, with company number 5030732, and is registered in the UK with company number FC030576 and branch number BR015634 at the address 1st Floor, 24 Hills Road, Cambridge CB2 1JP | August 2014
